# Non-HDL-C Is More Stable Than LDL-C in Assessing the Percent Attainment of Non-fasting Lipid for Coronary Heart Disease Patients

**DOI:** 10.3389/fcvm.2021.649181

**Published:** 2021-04-01

**Authors:** Li-Ling Guo, Yan-qiao Chen, Qiu-zhen Lin, Feng Tian, Qun-Yan Xiang, Li-yuan Zhu, Jin Xu, Tie Wen, Ling Liu

**Affiliations:** ^1^Department of Cardiovascular Medicine, The Second Xiangya Hospital, Central South University, Changsha, China; ^2^Research Institute of Blood Lipid and Atherosclerosis, Center South University, Changsha, China; ^3^Modern Cardiovascular Disease Clinical Technology Research Center of Hunan Province, Changsha, China; ^4^Cardiovascular Disease Research Center of Hunan Province, Changsha, China; ^5^Department of Emergency, The Second Xiangya Hospital, Central South University, Changsha, China

**Keywords:** coronary heart disease, non-fasting, low-density lipoprotein cholesterol, non-high-density lipoprotein cholesterol, cut-off points

## Abstract

This study aimed to compare the percentage attainment of fasting and non-fasting LDL-C and non-HDL-C target levels in coronary heart disease (CHD) patients receiving short-term statin therapy. This study enrolled 397 inpatients with CHD. Of these, 197 patients took statins for <1 month (m) or did not take any statin before admission (CHD1 group), while 204 patients took statins for ≥1 m before admission (CHD2 group). Blood lipid levels were measured at 0, 2, and 4 h after a daily breakfast. Non-fasting LDL-C and non-HDL-C levels significantly decreased after a daily meal (*P* < 0.05). Both fasting and non-fasting LDL-C or non-HDL-C levels were significantly lower in the CHD2 group. The percentage attainment of LDL-C <1.4 mmol/L at 2 and 4 h after a daily breakfast was significantly higher than that during fasting (*P* < 0.05), but the percent attainment of non-fasting non-HDL-C <2.2 mmol/L was close to its fasting value (*P* > 0.05). Analysis of c-statistic showed that non-fasting cut-off points for LDL-C and non-HDL-C were 1.19 and 2.11 mmol/L, corresponding to their fasting goal levels of 1.4 and 2.2 mmol/L, respectively. When post-prandial LDL-C and non-HDL-C goal attainments were re-evaluated using non-fasting cut-off points, there were no significant differences in percentage attainment between fasting and non-fasting states. Non-HDL-C is more stable than LDL-C in assessing the percent attainment of non-fasting lipid for coronary heart disease patients. If we want to use LDL-C to assess the percent attainment of post-prandial blood lipids, we may need to determine a lower non-fasting cut-off point.

## Introduction

Elevated cholesterol level is an independent risk factor for coronary heart disease (CHD). To reduce the risk of ischemic events for CHD patients, fasting level of low-density lipoprotein cholesterol (LDL-C) should be controlled to <1.4 mmol/L as the primary target, then that of non-high-density lipoprotein cholesterol (non-HDL-C) should be <2.2 mmol/L as the secondary target of cholesterol control according to the 2019 European guidelines (1).

It is increasingly believed that atherosclerosis is a post-prandial phenomenon because, at least with respect to lipids, we are in the post-prandial phase for the most part of the day (2). Considering that either fasting or non-fasting (i.e., post-prandial) LDL-C level has a similar predictive value for all-cause death and cardiovascular death (3, 4), non-fasting lipids detection at a random time-point within at least 8 h after a daily meal has been recommended in the primary and secondary prevention against CHD (5–8). However, both LDL-C and non-HDL-C levels show a tendency of decrease in the non-fasting state (9–11). Moreover, there are only fasting cholesterol-lowering targets but not non-fasting ones in the published guidelines (1, 12–15). It is uncertain whether these fasting targets are applicable to assessing cholesterol control as well as how to evaluate it in the non-fasting stage.

Recently, we observed more substantial reductions in LDL-C and non-HDL-C levels in Chinese subjects with CHD at 2 and 4 h after a daily breakfast (9, 16), appearing to be greater than those reported in large-scale clinical studies conducted in other countries (10, 11, 17, 18), although the potential cause remains uncertain. Additionally, it was proposed that non-HDL-C level may be a better prognostic factor than LDL-C level to evaluate the risk of future cardiovascular events (19–24). Furthermore, non-fasting fluctuation of non-HDL-C level seem to be smaller than that of LDL-C (24). Nevertheless, there have been no studies comparing the goal attainment of LDL-C with that of non-HDL-C in the non-fasting state.

Therefore, this study aimed to compare the percent attainments of fasting and non-fasting LDL-C and non-HDL-C reaching their fasting targets in CHD patients receiving short-term statins therapy. Furthermore, analysis of c-statistic or receiver operating characteristic curve (ROC) analysis was used to determine the non-fasting cut-off points corresponding to their fasting targets, and the percent attainments of non-fasting LDL-C and non-HDL-C were re-evaluated according to the non-fasting cut-off points.

## Materials and Methods

### Study Population

From March 2017 to July 2019, 397 inpatients with CHD were enrolled from the Department of Cardiovascular Medicine of the Second Xiangya Hospital, Central South University. A total of 84 patients did not take any statins and 109 patients took statins for <1 month (m) before admission (CHD1 group), and 204 patients took statins for ≥1 m before admission (CHD2 group). The definition of CHD was coronary atherosclerosis confirmed by coronary angiography and/or a history of myocardial infarction in patients with angina pectoris (AP). We asked all patients about their medical history and use of medication before enrollment. Patients with autoimmune disease, hepatic disorders, renal disease, cancer, or other serious diseases were excluded. The study was approved by the Ethics Committee of the Second Xiangya Hospital of Central South University. Informed consent was obtained from all patients.

### Specimen Collection

All enrolled participants ate breakfast between 7 a.m. and 8 a.m. according to their regular diets after overnight fast for at least 8 h. The patients in this study are all Han nationality, and their daily breakfast were rich in carbohydrate, including steamed bread, noodles, rice porridge, and so on, which were purchased from the cafeteria in our hospital or brought from home according to their dietary habits. During 4-h test, subjects were allowed only to drink a little water and walk slowly. Drinking wine or eating any food were not recommended.

### Determination of Blood Lipids Levels

Serum total cholesterol (TC) and triglyceride (TG) levels were measured using automated enzymatic assays. Serum high-density lipoprotein cholesterol (HDL-C) and LDL-C levels were measured using a chemical masking method (25). All measurements, including that of albumin, were carried out on a fully automatic biochemical analyser (Hitachi 7170A, Hitachi Inc., Tokyo, Japan) and performed by the expert who didn't know the details of the research (26). Non-HDL-C equals to TC minus HDL-C.

### Statistical Analysis

Data were analyzed using SPSS version 19.0. (IBM Corp., Armonk, NY, USA) and Prism 6.0 (GraphPad Inc., San Diego, CA, USA). Continuous variable values data were shown as mean ± standard deviation (SD), and categorical data were shown as numbers and percentages. The *t*-test and chi-square test were used to analyse continuous variables and categorical variables, respectively. The optimal cut-off points for fasting LDL-C (1.4 mmol/L) and non-HDL-C (2.2 mmol/L) were determined using receiver operating characteristic (ROC curve) analysis. Based on the ROC curve, values determined using Youden analysis were used as cut-off points. All tests were two-tailed, and *P* < 0.05 was considered statistically significant.

## Results

### Clinical Characteristics and Fasting Blood Lipids in Two CHD Groups

The baseline characteristics of the CHD patients are shown in Table 1. Both groups were similar in terms of age, sex, body mass index, percentages of hypertension, current smoking, and diabetes mellitus. There were 56.5% patients taking statins <1 m and 43.5% patients without statins treatment before admission in CHD1 group. Fasting serum levels of TC, LDL-C and non-HDL-C in CHD2 group were significantly lower than those in CHD1 group (*P* < 0.05). The differences in fasting serum TG and HDL-C levels between the groups did not differ significantly (Table 1). The proportion of STEMI and NSTEMI patients in CHD2 group was less than that in CHD1 group (*P* < 0.05). However, the proportion of schemic cardiomyopathy in CHD2 group was more than that in CHD1 group (*P* < 0.05). The differences in vascular disease (single or multiple vessel disease) did not differ significantly (Table 1).

**Table 1:** Baseline characteristics of the study population.

	**CHD1 (*n* = 193)**	**CHD2 (*n* = 204)**
Age (y, SD)	60.3 ± 9.3	62.0 ± 8.7
Men, *n* (%)	156 (80.8)	157 (77.0)
BMI (kg/m^2^, SD)	24.5 ± 3.5	24.9 ± 3.0
Hypertension, *n* (%)	141 (73.1)	152 (74.5)
Current smoking, *n* (%)	110 (57.0)	103 (50.5)
DM, *n* (%)	51 (26.4)	67 (32.8)
Taking statins, *n* (%)		
Statins ≥1 m	0	204 (100)^*^
Statins <1 m	109 (56.5)	0^*^
No statins	84 (43.5)	0^*^
Vascular disease, *n* (%)		
Single vessel disease	43 (22.3)	36 (17.6)
Multiple vessel disease	132 (68.4)	146 (71.6)
Without CAG	18 (9.3)	22 (10.8)
CHD subtype, *n* (%)		
STEMI	14 (7.3)	4 (2.0)^*^
NSTEMI	32 (16.6)	13 (6.4)^*^
UA	102 (52.8)	114 (55.9)
SAP	31 (16.1)	42 (20.6)
Schemic cardiomyopathy	4 (2.1)	16 (7.8)^*^
Others	10 (5.2)	15 (7.4)
TC (mmol/L, SD)	4.32 ± 0.91	3.93 ± 1.03^*^
LDL-C (mmol/L, SD)	2.72 ± 0.79	2.43 ± 0.90^*^
Non-HDL-C (mmol/L, SD)	3.20 ± 0.96	2.92 ± 0.98^*^
HDL-C (mmol/L, SD)	1.12 ± 0.27	1.01 ± 0.25
TG (mmol/L, SD)	1.74 ± 1.11	1.84 ± 1.29

*CHD1 group: CHD patients taking statins <1 m and without statins treatment before admission. CHD2 group: CHD patients taking statins ≥1 m before admission. BMI, body mass index; DM, diabetes mellitus; SAP, stable angina pectoris; Others in CHD subtype: Including coronary microangiopathy, etc. TG, triglyceride; TC, total cholesterol; HDL-C, high-density lipoprotein cholesterol; LDL-C, low-density lipoprotein. Continuous variable values were reported as mean ± SD, and categorical data were reported as numbers and percentages*.

* *P < 0.05 when compared with CHD1 group*.

According to the time of statin using before admission, all patients were divided into three groups: CHD1 group (non-statin taking, *n* = 93), CHD2 group [short-term statin taking (<1 m), *n* = 109] and CHD3 group [long-term statin taking (≥1 m), *n* = 204], which were added as the supplement data in the new manuscript due to the small sample size in this study (Supplementary Table 1).

### Comparison of Changes in Non-fasting Blood Lipids in the Two Groups

**Figure 1:**
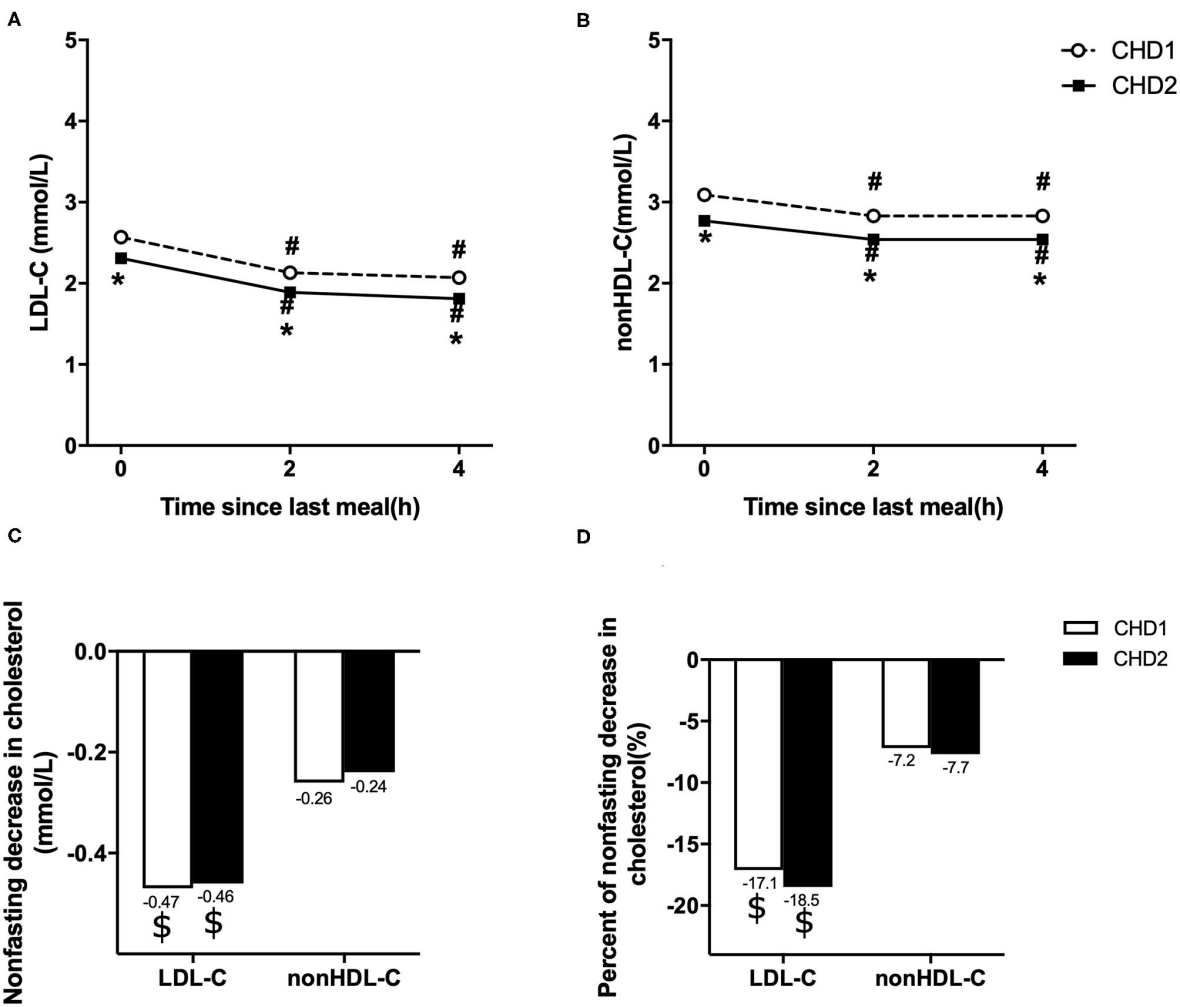
Comparison of changes in non-fasting blood lipids in two groups. Non-fasting changes in serum levels of LDL-C **(A)** and non-HDL-C **(B)**. Values are mean ± Standard Error (SE). Comparison of non-fasting absolute reductions in LDL-C and non-HDL-C levels **(C)**. Comparison of non-fasting percentages of reductions in LDL-C and non-HDL-C levels **(D)**. CHD1 group: CHD patients taking statins <1 m and without statins treatment before admission. CHD2 group: CHD patients taking statins ≥1 m before admission. ^#^*P* < 0.05 when compared with the fasting level in the same group. *P < 0.05 when compared with CHD1 group at the same time point. ^$^*P* < 0.05 when compared with the absolute reduction or percentage of reduction in non-HDL-C level.

Levels of LDL-C and non-HDL-C decreased significantly at 2 and 4 h after a daily breakfast (*P* < 0.05). Non-fasting LDL-C and non-HDL-C in CHD2 group were significantly lower than those in CHD1 group (*P* < 0.05; Figures 1A,B). When the data at 2 and 4 h after a daily meal as a whole were used as non-fasting data for further analysis, non-fasting reductions in LDL-C were 0.47 and 0.46 mmol/L, and non-HDL-C were 0.26 and 0.24 mmol/L in CHD1 and CHD2 groups, respectively (Figure 1C). The percentages of reduction in LDL-C were 17.1 and 18.5%, and 7.2 and 7.7% in non-HDL-C in CHD1 and CHD2 groups, respectively (Figure 1D). There were no significant differences in the absolute reduction or percentage of reduction in LDL-C or non-HDL-C level between the groups. However, non-fasting reductions in LDL-C were greater than those of non-HDL-C (Figure 1B).

Non-fasting albumin levels were measured in 89 patients among all CHD patients. There was no significant change in the albumin level after a daily breakfast (Table 2), whereas the post-prandial TC, HDL-C, LDL-C, and non-HDL-C level dropped significantly (*P* < 0.05; Table 2).

**Table 2:** Changes in levels of blood lipids and albumin after a daily breakfast in 89 CHD patients.

	**Fasting**	**2 h after meal**	**4 h after meal**
TG (mmol/L, SD)	2.09 ± 1.93	2.34 ± 1.88	2.63 ± 2.19
TC (mmol/L, SD)	4.02 ± 1.07	3.72 ± 0.96^*^	3.71 ± 0.96^*^
HDL-C (mmol/L, SD)	1.03 ± 0.22	1.01 ± 0.21^*^	1.03 ± 0.28^*^
LDL-C (mmol/L, SD)	2.51 ± 0.95	2.11 ± 0.71^*^	2.07 ± 0.74^*^
Non-HDL-C (mmol/L, SD)	2.99 ± 1.10	2.71 ± 0.89^*^	2.69 ± 0.97^*^
Albumin (g/L, SD)	38.3 ± 2.85	38.3 ± 3.07	38.5 ± 3.00

*TG, triglyceride; TC, total cholesterol; HDL-C, high-density lipoprotein cholesterol; LDL-C, low-density lipoprotein. CHD patients including patients in CHD1 group and CHD2 group. Data are mean ± SD*.

* *P < 0.05 when compared with fasting state*.

### Evaluating Goal Attainments of LDL-C and Non-HDL-C According to Various Targets in CHD2 Group

**Figure 2:**
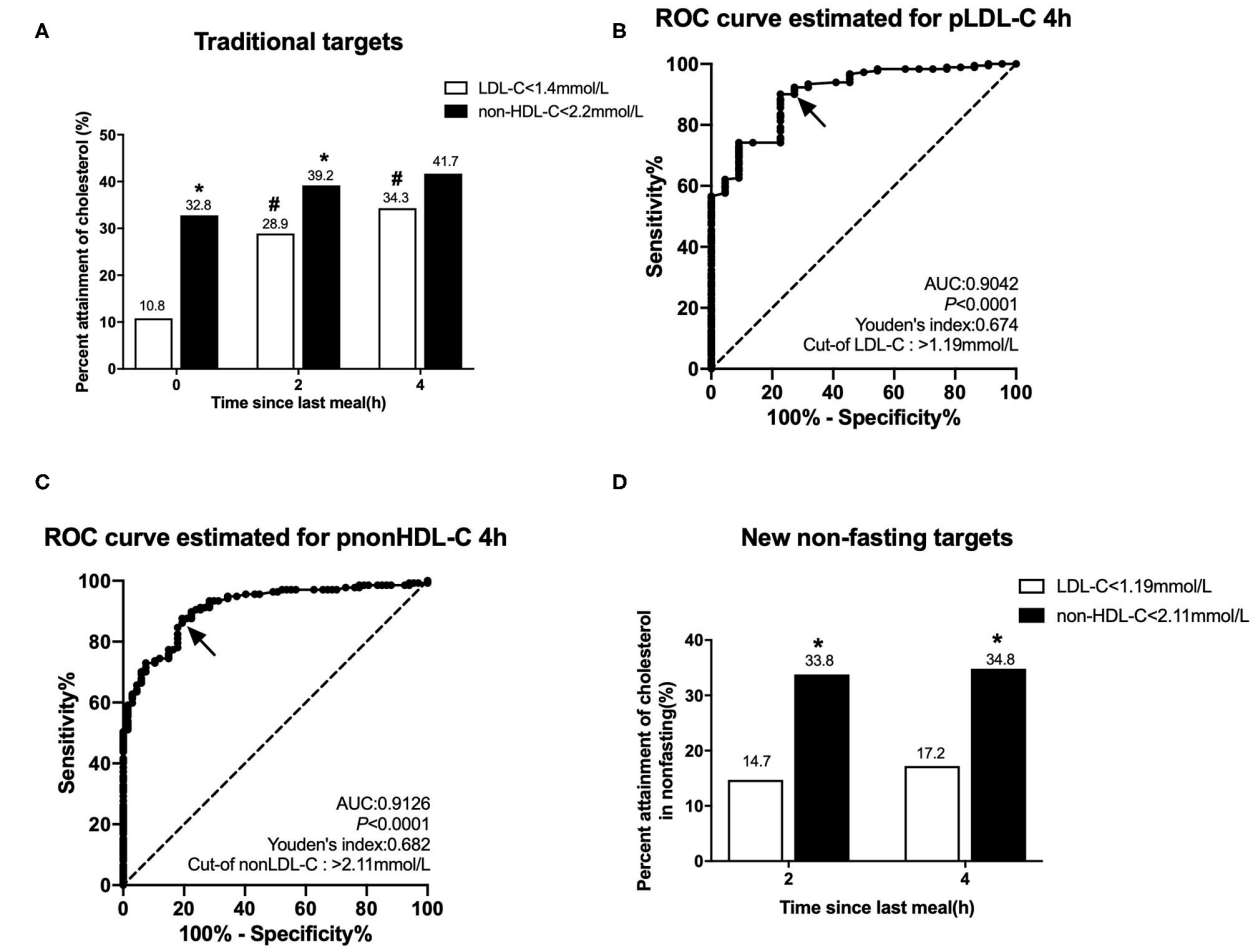
Comparison of the percent attainments of LDL-C and non-HDL-C levels according to different targets in fasting and non-fasting states in Group CHD2. CHD2 group: CHD patients taking statins ≥1 m before admission. **(A)** Comparison of the percent attainments in both fasting and non-fasting states according to the recommended targets of LDL-C level <1.4 mmol/L and non-HDL-C level <2.2 mmol/L. **(B,C)** Non-fasting cut-off points in relation to fasting LDL-C level 1.4 mmol/L **(B)** and non-HDL-C level 2.2 mmol/L **(C)** determined by ROC analysis and Youden's index (marked by the solid arrows). **(D)** Comparison of the percent attainments according to non-fasting cut-off points of LDL-C <1.19 mmol/L and non-HDL-C <2.11 mmol/L. ^#^*P* < 0.05 when compared with the percent attainment of the same target(s) in the fasting state. *P < 0.05 when compared with the percent attainment of different LDL-C target at the same time point.

According to the 2019 European guidelines (1), the percent attainment of LDL-C <1.4 mmol/L in the fasting state was significantly lower than that of non-HDL-C <2.2 mmol/L (10.8 vs. 32.8%, *P* < 0.001). After a daily breakfast, the percent attainment of non-HDL-C and LDL-C gradually tightened, and there was statistical difference only at 2 h. The percent attainment of LDL-C at 2 or 4 h was significantly higher than its fasting value (*P* < 0.05), but there is no difference in non-HDL-C between fasting and post-prandial values (*P* > 0.05; Figure 2A).

ROC curve analysis showed that non-fasting cut-off points for LDL-C and non-HDL-C at 4 h were 1.19 mmol/L (sensitivity 90.1%, specificity 77.3%, and AUC 0.904) and 2.11 mmol/L (sensitivity 87.6%, specificity 80.6%, and AUC 0.913), corresponding to their fasting goal levels of 1.4 and 2.2 mmol/L, respectively (Figures 2B,C).

According to the non-fasting cut-off points, the percent attainment of LDL-C <1.19 mmol/L at 2 or 4 h was 14.7 or 17.2%, which was close to the percent attainment of LDL-C <1.4 mmol/L in the fasting state. The percent attainment of non-HDL-C <2.11 mmol/L at 2 or 4 h was close to the percent attainment of non-HDL-C <2.2 mmol/L in the fasting state. Moreover, the percent attainment of LDL-C <1.19 mmol/L was significantly lower than that of non-HDL-C <2.11 mmol/L at 2 or 4 h (Figure 2D).

## Discussion

In this study, we found that when LDL-C goal <1.4 mmol/L was used for evaluating cholesterol control in Chinese CHD patients after short-term statins treatment, the target percentage attainment in the non-fasting state was significantly higher than that of the fasting state. However, the percent attainment of non-fasting non-HDL-C was close to its fasting state, suggesting that non-HDL-C is more stable than LDL-C in assessing the percent attainment of non-fasting lipid for coronary heart disease patients. Notably, according to the new non-fasting cut-off points, 1.19 mmol/L, the non-fasting goal attainment of LDL-C was close to its fasting value. This suggests that lower non-fasting targets could be needed to evaluate the efficacy of cholesterol-lowing therapy in the non-fasting state, particularly when fasting blood lipids are unavailable and the percentage reduction of LDL-C cannot be determined due to a lack of baseline non-fasting levels before treatment.

There are two targets to evaluate the efficacy of cholesterol-lowering treatment in CHD patients. First, LDL-C should achieve a ≥50% reduction from baseline or a goal <1.4 mmol/L according to the 2019 European guidelines (1). However, this recommendation refers only to cholesterol control in the fasting state. In this study, the goal attainment of LDL-C reduction ≥50% could not be evaluated because the baseline fasting or non-fasting LDL-C levels before treatment could not be obtained in most patients in the CHD2 group. Under these circumstances, a physician can only make clinical judgments based on LDL-C goal levels. A considerable number of CHD patients from other locations visit physicians but forget to remain in a fasting state. This is a common situation in the outpatient department of our hospital. As a result, physicians have to assess cholesterol control using non-fasting measurement of blood lipids. According to the joint consensus statement of European Atherosclerosis Society and European Federation of Clinical Chemistry and Laboratory Medicine (5), the non-fasting detection of blood lipids can be routinely applied in CHD patients as long as they are willing to undergo non-fasting measurement. This suggests that measurement of LDL-C level in the non-fasting state is quite important.

Compared with some studies with large population in other countries (10, 11, 17, 18), the reduction in LDL-C level in CHD patients after a daily meal was more significant in the present study. The maximum mean reduction in LDL-C or non-HDL-C was ~0.1–0.2 mmol/L in the European and North American subjects (10, 11, 17, 18); however, Chinese CHD patients in this study showed a greater decrease in either directly detected LDL-C (i.e., 0.4–0.5 mmol/L) or calculated non-HDL-C (i.e., 0.2–0.3 mmol/L) after a daily breakfast. Although our recent study showed that the post-prandial decline (i.e., 0.3–0.4 mmol/L) in calculated LDL-C was less than that of the directly detected LDL at 2–4 h after a daily breakfast in Chinese CHD patients (9), it was still more than the reduction of above the European and North American studies (10, 11, 17, 18). The underling mechanisms of non-fasting reduction in LDL-C may be complicated in the present study. First, in the Copenhagen General Population Study, they compared blood lipids levels of individuals at random time points after the last meal in the large-scale population. By contrast, our measurements were acquired from the same individuals at various times since the last meal, which was different from the Copenhagen General Population Study in terms of the observation time-points and monitoring method. Second, post-prandial reduction in LDL-C concentration is most likely haemodilution resulting from fluid intake in relation to the meal and thus adjusting the data for albumin concentration was recommended (7, 10). Langsted et al. (10) observed that the non-fasting LDL-C concentration no longer changed after adjustment for albumin concentration. However, a very slight change in the post-prandial albumin level was observed in our study; therefore, haemodilution may not be the only cause of post-prandial decline in the LDL-C level in the Chinese. Third, the diet structures of Chinese and western people are very different. For example, the Chinese people prefer carbohydrates (16). It is not clear whether the high-carbohydrate diet will cause a more significant decline in cholesterol. At any rate, the obvious decrease in non-fasting LDL-C might affect the evaluation of goal attainment when the LDL-C level was detected after a meal.

Indeed, non-HDL-C was more stable than LDL-C in assessing the percent attainment of non-fasting lipid for coronary heart disease patients. Non-fasting reduction in non-HDL-C was less than that in LDL-C and the difference between fasting and non-fasting percentage attainments of non-HDL-C <2.2 mmol/L was less than that of LDL-C <1.4 mmol/L. Non-HDL-C represents the cholesterol content of all atherosclerotic lipoproteins in the circulation, including chylomicrons, very-low-density-lipid and their remnants, intermediate-density lipoproteins, LDL, and lipoprotein (a) particles. Takahiro found that non-HDL cholesterol levels were clearly associated with future mortality and were less affected by fasting status or serum triglyceride levels (27). Meta-analyses and prospective studies with large populations supported the opinion that on-treatment levels of non-HDL-C were stronger than that of LDL-C for future CVD risk estimation (20, 21). Furthermore, non-HDL-C is a cheaper equivalent predictor of risk on and off statins, without the requirement for a fasting sample (28). Therefore, some scholars proposed that the clinical benefit obtained from controlling non-HDL-C would be greater than the one obtained from controlling LDL-C (19–21, 23).

Nevertheless, the percent attainment of non-HDL-C was higher than that of LDL-C in both fasting and non-fasting states according to the goals of LDL-C <1.4 mmol/L and non-HDL-C <2.2 mmol/L, respectively, in this study. The difference between non-HDL-C and LDL-C will increase with TG elevation, which could exert a substantial influence on evaluation of cholesterol-lowering treatment (29, 30). The fixed difference between fasting non-HDL-C and LDL-C goals was 30 mg/dl (i.e., 0.8 mmol/L) when fasting TG level was 1.7 mmol/L, reflecting the fact that cholesterol content within TG-rich lipoproteins was about 1.7/2.2 ≈ 0.8 mmol/L. Some scholars found that the goal attainment of non-HDL-C was higher than that of LDL-C when fasting TG was <1.7 mmol/L, while it was less than that of LDL-C when fasting TG >2.3 mmol/L (29). Su et al. reported that the specific and fixed goals as non-HDL-C 0.8 mmol/L (30 mg/dL) higher than the corresponding LDL-C goals were not sufficient for Chinese patients with CHD and proposed that flexible goals basing on TG level were more appropriate (30). This is consistent with our findings that the percent attainment of LDL-C <1.4 mmol/L was significantly lower than that of non-HDL-C <2.2 mmol/L in the fasting state; however, the difference in percent attainment between LDL-C and non-HDL-C after a daily breakfast became smaller with the increase in non-fasting TG level.

It was found that the percent attainment of post-prandial LDL-C was significantly higher than that of fasting values in the present study, suggesting that the fasting goals of LDL-C <1.4 mmol/L was indeed unsuitable for the evaluation of post-prandial cholesterol control. ROC analysis has been used to identify the optimal cut-off point for the diagnosis of post-prandial hypertriglyceridemia (16, 31, 32) but not for determining goals of LDL-C and non-HDL-C in the non-fasting state corresponding to the fasting goals. Because the non-fasting cut-off points acquired by ROC analysis corresponded to the fasting goals of LDL-C <1.4 mmol/L and non-HDL-C <2.2 mmol/L, the post-prandial percent attainments were very similar to their respective fasting values. This suggested that lower post-prandial cut-off points, different from their fasting goals, should be adopted in the evaluation of post-prandial goal attainment, unless it is possible to assess the percentage reduction in the non-fasting LDL-C level. In this study, the difference (1.19 vs. 2.11 mmol/L) between non-fasting cut-off points of LDL-C and non-HDL-C was 0.92 mmol/L corresponding to non-fasting TG level of ~2.0 mmol/L (i.e., 0.92 × 2.2 = 2.024 ≈ 2.0). This suggests that a larger difference between LDL-C and non-HDL-C should be considered in the evaluation of non-fasting goal attainment even after a daily meal without high fat.

This study had some limitations. First, it was a single center study with a small sample size of inpatients. In the future, the suitability of non-fasting cut-off points in a large sample of arteriosclerotic cardiovascular disease patients, including patients with ischemic stroke and peripheral vascular disease, is worth exploring. Second, only the percent attainment of the goal, but not percentage reduction of LDL-C, was evaluated because of the lack of baseline levels of blood lipids. Third, it can only reflect the situation of patients admitted to the hospital. There are also a large number of outpatients with relatively stable conditions and their medication situation may be different from that of hospitalized patients. Therefore, the changes of post-prandial blood lipid may be different from our study. This needs further study in the future.

## Conclusions

Non-HDL-C is more stable than LDL-C in assessing the percent attainment of non-fasting lipid for coronary heart disease patients. If we want to use LDL-C to assess the percent attainment of post-prandial blood lipids, we may need to determine a lower non-fasting cut-off point.

## Data Availability Statement

The original contributions generated for the study are included in the article/Supplementary Material, further inquiries can be directed to the corresponding author/s.

## Ethics Statement

Written informed consent was obtained from the individual(s) for the publication of any potentially identifiable images or data included in this article.

## Author Contributions

L-LG, Y-qC, and LL designed and conducted of this study. Q-zL, FT, Q-YX, and L-yZ participated in the collection, analysis, and interpretation of the data. L-LG and TW contributed to the preparation of the manuscript. LL carried out the approval of the study. All authors read the study and approved the manuscript for publication.

## Conflict of Interest

The authors declare that the research was conducted in the absence of any commercial or financial relationships that could be construed as a potential conflict of interest.

## References

[1] Mach F Baigent C Catapano AL Koskinas KC Casula M Badimon L. 2019 ESC/EAS Guidelines for the management of dyslipidaemias: lipid modification to reduce cardiovascular risk. Eur Heart J. (2019) 41:111–8. 10.15829/1560-4071-2020-3826 31504418

[2] Pastromas S Terzi AB Tousoulis D Koulouris S. Postprandial lipemia: an under-recognized atherogenic factor in patients with diabetes mellitus. Int J Cardiol. (2008) 126:3–12. 10.1016/j.ijcard.2007.04.172 17689745

[3] Doran B Guo Y Xu J Weintraub H Mora S Maron DJ. Prognostic value of fasting versus nonfasting low-density lipoprotein cholesterol levels on long-term mortality: insight from the National Health and Nutrition Examination Survey III (NHANES-III). Circulation. (2014) 130:546–53. 10.1161/CIRCULATIONAHA.114.010001 25015340

[4] Langsted A Nordestgaard BG. Nonfasting versus fasting lipid profile for cardiovascular risk prediction. Pathology. (2019) 51:131–41. 10.1016/j.pathol.2018.09.062 30522787

[5] Nordestgaard BG Langsted A Mora S Kolovou G Baum H Bruckert E. Fasting is not routinely required for determination of a lipid profile: clinical and laboratory implications including flagging at desirable concentration cut-points-a joint consensus statement from the European Atherosclerosis Society and European Federation of Clinical Chemistry and Laboratory Medicine. Eur Heart J. (2016) 37:1944–58. 10.1093/eurheartj/ehw152 27122601 PMC4929379

[6] Steen DL Khan I Ansell D Sanchez RJ Ray KK. Retrospective examination of lipid-lowing treatment patterns in a real-world high-risk cohort in the UK in 2014: comparison with the National Institute for Health and Care Excellence (NICE) 2014 lipid modification guidelines. BMJ Open. (2017) 7:e013255. 10.1136/bmjopen-2016-013255 PMC5318572 28213597

[7] Langsted A Nordestgaard BG. Nonfasting lipids, lipoproteins, and apolipoproteins in individuals with and without diabetes: 58 434 individuals from the Copenhagen General Population Study. Clin Chem. (2011) 57:482–9. 10.1373/clinchem.2010.157164 21189274

[8] Downs JR O'Malley PG. Management of dyslipidemia for cardiovascular disease risk reduction: synopsis of the 2014. U.S. Department of Veterans Affairs and U.S. Department of Defense clinical practice guideline. Ann Intern Med. (2015) 163:291–7. 10.7326/M15-0840 26099117

[9] Lin QZ Chen YQ Guo LL Xiang QY Tian F Wen T. Comparison of non-fasting LDL-C levels calculated by Friedewald formula with those directly measured in Chinese patients with coronary heart disease after a daily breakfast. Clin Chim Acta. (2019) 495:399–405. 10.1016/j.cca.2019.05.010 31085187

[10] Langsted A Freiberg JJ Nordestgaard BG. Fasting and nonfasting lipid levels: influence of normal food intake on lipids, lipoproteins, apolipoproteins, and cardiovascular risk prediction. Circulation. (2008) 118:2047–56. 10.1161/CIRCULATIONAHA.108.804146 18955664

[11] Mora S Rifai N Buring JE Ridker PM. Fasting compared with nonfasting lipids and apolipoproteins for predicting incident cardiovascular events. Circulation. (2008) 118:993–1001. 10.1161/CIRCULATIONAHA.108.777334 18711012 PMC2574817

[12] Stone NJ Robinson JG Lichtenstein AH Bairey Merz CN Blum CB Eckel RH. 2013 ACC/AHA guideline on the treatment of blood cholesterol to reduce atherosclerotic cardiovascular risk in adults: a report of the American College of Cardiology/American Heart Association Task Force on Practice Guidelines. J Am Coll Cardiol. (2014). 63:2889–934. 10.1161/01.cir.0000437738.63853.7a 24239923

[13] Joint committee for guideline revision National Expert Committee on Cardiovascular Diseases, National Center for Cardiovascular Diseases Chinese Society of Cardiology, Chinese Medical Association Chinese Diabetes Society, Chinese Medical Association Chinese Society of Endocrinology, Chinese Medical Association Chinese Society of laboratory Medicine, Chinese Medical Association Writing Group Members Group Leader. 2016 Chinese guidelines for the management of dyslipidemia in adults. J Geriatr Cardiol. (2018). 15:1–29. 10.11909/j.issn.1671-5411.2018.01.011 29434622 PMC5803534

[14] Catapano AL Graham I De Backer G Wiklund O Chapman MJ Drexel H. 2016 ESC/EAS guidelines for the management of dyslipidaemias. Eur Heart J. (2016) 37:2999–3058. 10.1093/eurheartj/ehw272 27567407

[15] Grundy SM Stone NJ Bailey AL Beam C Birtcher KK Blumenthal RS. 2018 AHA/ACC/AACVPR/AAPA/ABC/ACPM/ADA/AGS/APhA/ASPC/NLA/PCNA Guideline on the Management of Blood Cholesterol: A Report of the American College of Cardiology/American Heart Association Task Force on Clinical Practice Guidelines. J Am Coll Cardiol. (2019). 73:e285–350. 10.1016/j.jacc.2018.11.004 30423393

[16] Tian F Xiang QY Zhang MY Chen YQ Lin QZ Wen T. Changes in non-fasting concentrations of blood lipids after a daily Chinese breakfast in overweight subjects without fasting hypertriglyceridemia. Clin Chim Acta. (2019) 490:147–53. 10.1016/j.cca.2019.01.004 30615853

[17] Sidhu D Naugler C. Fasting time and lipid levels in a community-based population: a cross-sectional study. Arch Intern Med. (2012) 172:1707–10. 10.1001/archinternmed.2012.3708 23147400

[18] Bansal S Buring JE Rifai N Mora S Sacks FM Ridker PM. Fasting compared with nonfasting triglycerides and risk of cardiovascular events in women. JAMA. (2007) 298:309–16. 10.1001/jama.298.3.309 17635891

[19] Benn M. Apolipoprotein B levels, APOB alleles, and risk of ischemic cardiovascular disease in the general population, a review. Atherosclerosis. (2009) 206:17–30. 10.1016/j.atherosclerosis.2009.01.004 19200547

[20] Toth PP. Association of LDL cholesterol, non–HDL cholesterol, and apolipoprotein B levels with risk of cardiovascular events among patients treated with statins: a meta-analysis. Yearbook Endocrinol. (2012) 2012:65–8. 10.1016/j.yend.2012.05.028 22453571

[21] Ridker PM Rifai N Cook NR Bradwin G Buring JE. Non-HDL cholesterol, apolipoproteins A-I and B100, standard lipid measures, lipid ratios, and CRP as risk factors for cardiovascular disease in women. JAMA. (2005) 294:326–33. 10.1001/jama.294.3.326 16030277

[22] Di Angelantonio E Gao P Pennells L Kaptoge S Caslake M Thompson A. Lipid-related markers and cardiovascular disease prediction. JAMA. (2012) 307:2499–506. 10.1001/jama.2012.6571 22797450 PMC4211641

[23] Carbayo Herencia JA Simarro Rueda M Palazon Bru A Molina Escribano F Ponce Garcia I Artigao Rodenas LM. Evaluation of non-HDL cholesterol as a predictor of non-fatal cardiovascular events in a prospective population cohort. Clin Investig Arterioscler. (2018) 30:64–71. 10.1016/j.artere.2017.10.003 29395492

[24] de Vries M Klop B Castro Cabezas M. The use of the non-fasting lipid profile for lipid-lowering therapy in clinical practice - point of view. Atherosclerosis. (2014) 234:473–5. 10.1016/j.atherosclerosis.2014.03.024 24814412

[25] Zhao Y Peng R Zhao W Liu Q Guo Y Zhao S. Zhibitai and low-dose atorvastatin reduce blood lipids and inflammation in patients with coronary artery disease. Medicine. (2017) 96:e6104. 10.1097/MD.0000000000006104 28207527 PMC5319516

[26] Tian F Wu CL Yu BL Liu L Hu JR. Apolipoprotein O expression in mouse liver enhances hepatic lipid accumulation by impairing mitochondrial function. Biochem Biophys Res Commun. (2017) 491:8–14. 10.1016/j.bbrc.2017.06.128 28647361

[27] Ito T Arima H Fujiyoshi A Miura K Takashima N Ohkubo T. Relationship between non-high-density lipoprotein cholesterol and the long-term mortality of cardiovascular diseases: NIPPON DATA 90. Int J Cardiol. (2016) 220:262–7. 10.1016/j.ijcard.2016.06.021 27389451

[28] Welsh C Celis-Morales CA Brown R Mackay DF Lewsey J Mark PB. Comparison of conventional lipoprotein tests and apolipoproteins in the prediction of cardiovascular disease. Circulation. (2019) 140:542–52. 10.1161/CIRCULATIONAHA.119.041149 31216866 PMC6693929

[29] Al-Hashmi K Al-Zakwani I Al Mahmeed W Arafah M Al-Hinai AT Shehab A. Non-high-density lipoprotein cholesterol target achievement in patients on lipid-lowering drugs and stratified by triglyceride levels in the Arabian Gulf. J Clin Lipidol. (2016) 10:368–77. 10.1016/j.jacl.2015.12.021 27055968

[30] Su X Luo M Tang X Luo Y Zheng X Peng D. Goals of non-high density lipoprotein cholesterol need to be adjusted in Chinese acute coronary syndrome patients: findings from the CCC-ACS project. Clin Chim Acta. (2019) 496:48–54. 10.1016/j.cca.2019.06.022 31255567

[31] White KT Moorthy MV Akinkuolie AO Demler O Ridker PM Cook NR. Identifying an optimal cutpoint for the diagnosis of hypertriglyceridemia in the nonfasting state. Clin Chem. (2015) 61:1156–63. 10.1373/clinchem.2015.241752 26071491 PMC4554926

[32] Sevilla-Gonzalez MDR Aguilar-Salinas CA Munoz-Hernandez L Almeda-Valdes P Mehta R Zubiran R. Identification of a threshold to discriminate fasting hypertriglyceridemia with postprandial values. Lipids Health Dis. (2018) 17:156. 10.1186/s12944-018-0803-8 30021651 PMC6052549

