# Supplementary material for: Non-HDL-C Is More Stable Than LDL-C in Assessing the Percent Attainment of Non-fasting Lipid for Coronary Heart Disease Patients
Source: Front Cardiovasc Med. 2021 Apr 1;8:649181. doi: 10.3389/fcvm.2021.649181 (PMC8049565; doi:10.3389/fcvm.2021.649181)
Supplement: Supplementary file 1 [file Table_1.docx]

Supplementary table 1. Baseline characteristics of the study population.

|  | CHD1  (n=84) | CHD2  (n=109) | | CHD3  (n=204) |
| --- | --- | --- | --- | --- |
| Age (y, SD)  Men (n, %)  BMI (kg/m2, SD)  Hypertension (n, %)  Current smoking (n, %)  DM (n, %)  Vascular disease (n, %)  single vessel disease  multiple vessel disease  Without CAG  CHD subtype (n, %)  STEMI  NSTEMI  UA  SAP  Ischemic cardiomyopathy  Others  TC (mmol/L, SD)  LDL-C (mmol/L, SD)  Non-HDL-C (mmol/L, SD)  HDL-C (mmol/L, SD)  TG (mmol/L, SD) | 59.5 ± 9.6  67 (79.8)  24.9 ± 3.1  61 (72.6)  46 (54.8)  24 (28.5)  24 (28.6)  49 (58.3)  11 (13.1)  9 (10.7)  12 (14.3)  45 (53.6)  12 (14.3)  1 (1.2)  5 (6.0)  4.40 ± 1.06  2.80 ± 0.92  3.35 ± 0.96  1.05 ± 0.28  1.93 ± 1.27 | 60.9 ± 9.0  89 (81.7)  24.8 ± 2.8  80 (73.4)  64 (58.7)  27 (24.8)  19 (17.4)  83 (76.1)  7 (6.4)  5 (4.6)  20 (18.3)  57 (52.3)  19 (17.4)  3 (2.8)  5 (4.6)  3.86 ± 0.96*  2.39 ± 0.84*  2.89 ± 0.92*  0.97 ± 0.23  1.95 ± 1.60 | 62.0 ± 8.7  157 (76.9)  24.9 ± 3.0  152 (74.5)  103 (50.5)  67 (32.8)  36 (17.6)  146 (71.6)  22 (10.8)  4 (2.0) *  13 (6.4) ^&^  114 (55.9)  42 (20.6)  16 (7.8)  15 (7.4)  3.78 ± 1.0*  2.31 ± 0.88*  2.77 ± 0.97*  1.00 ± 0.24  1.74 ± 1.10 | |

CHD1 group: CHD patients non taking statin before admission；CHD2 group: CHD patients taking statins <1m before admission. CHD3 group: CHD patients taking statins ≥1m before admission. BMI, body mass index; DM, diabetes mellitus; SAP: stable angina pectoris; Others in CHD subtype: Including coronary microangiopathy, ect. TG, triglyceride; TC, total cholesterol; HDL-C, high-density lipoprotein cholesterol; LDL-C, low-density lipoprotein. Continuous variable values were reported as mean ± SD, and categorical data were reported as numbers and percentages. *P<0 .05 when compared with CHD1 group. ^&^ P<0 .05 when compared with CHD2 group.
